# Circulating levels of endotrophin and cross-linked type III collagen reflect liver fibrosis in people with HIV

**DOI:** 10.1186/s12879-023-08000-w

**Published:** 2023-01-24

**Authors:** Leona Dold, Mette J. Nielsen, Michael Praktiknjo, Carolynne Schwarze-Zander, Christoph Boesecke, Jan-Christian Wasmuth, Jenny Bischoff, Jürgen Kurt Rockstroh, Morten A. Karsdal, Ulrich Spengler, Jonel Trebicka, Christian P. Strassburg, Diana J. Leeming, Bettina Langhans

**Affiliations:** 1 Department of Internal Medicine I, University Hospital of Bonn, Venusberg-Campus 1, 53127 Bonn, Germany; 2 Nordic Bioscience, Fibrosis Biology and Biomarkers, Herlev, Denmark; 3 German Center for Infection Research (DZIF), Partner Site Bonn-Cologne, Bonn, Germany; 4 Department of Internal Medicine B, University Hospital of Münster, Münster, Germany; 5 European Foundation for the Study of Chronic Liver Failure (EF-CLIF), Barcelona, Spain; 6 Faculty of Health Sciences, University of Southern Denmark, Odense, Denmark

**Keywords:** PC3X, PRO-C5, PRO-C6, HIV, Liver fibrosis, Hepatic steatosis, Endotrophin

## Abstract

**Background and aims:**

Liver-associated complications still frequently lead to mortality in people with HIV (PWH), even though combined antiretroviral treatment (cART) has significantly improved overall survival. The quantification of circulating collagen fragments released during collagen formation and degradation correlate with the turnover of extracellular matrix (ECM) in liver disease. Here, we analysed the levels of ECM turnover markers PC3X, PRO-C5, and PRO-C6 in PWH and correlated these with hepatic fibrosis and steatosis.

**Methods:**

This monocentre, retrospective study included 141 PWH. Liver stiffness and liver fat content were determined using transient elastography (Fibroscan) with integrated CAP function. Serum levels of formation of cross-linked type III collagen (PC3X), formation of type V collagen (PRO-C5) and formation type VI collagen (PRO-C6), also known as the hormone endotrophin, were measured with ELISA.

**Results:**

Twenty-five (17.7%) of 141 PWH had clinical significant fibrosis with liver stiffness ≥ 7.1 kPa, and 62 PWH (44.0%) had steatosis with a CAP value > 238 dB/m. Study participants with fibrosis were older (p = 0.004) and had higher levels of AST (p = 0.037) and lower number of thrombocytes compared to individuals without fibrosis (p = 0.0001). PC3X and PRO-C6 were markedly elevated in PWH with fibrosis. Multivariable cox regression analysis confirmed PC3X as independently associated with hepatic fibrosis. PRO-C5 was significantly elevated in participants with presence of hepatic steatosis.

**Conclusion:**

Serological levels of cross-linked type III collagen formation and endotrophin were significantly associated with liver fibrosis in PWH receiving cART and thus may be suitable as a non-invasive evaluation of liver fibrosis in HIV disease.

**Supplementary Information:**

The online version contains supplementary material available at 10.1186/s12879-023-08000-w.

## Introduction

Treatment of PWH with combined antiretroviral therapy (cART) has sustainably reduced death rates from opportunistic diseases. However, PWH still have high risk of developing non-alcoholic fatty liver disease (NAFLD) and liver fibrosis possibly leading to cirrhosis and liver cancer [1–3]. Antiretroviral treatment is overall liver-friendly with few liver-related adverse effects and leads to an overall improvement in fibrogenesis [4–9]. Besides the overwhelming positive effect, it was shown that cART induces oxidative stress and thus could contribute to liver dysfunction in virally controlled PWH [10, 11]. HIV itself could cause liver injury due to altered lipid metabolism, and impaired gut mucosa can lead to accumulation of bacterial products in the portal circulation [12, 13].

Synthesis and degradation of the extracellular matrix (ECM) are increased during progression of liver fibrosis [14–16]. ECM is degraded by matrix metalloproteinases (MMPs), so that small collagen fragments are formed which can ultimately be measured in the person’s serum and enable to determine the extent of ECM turnover [18]. Several collagen neo-epitope fragments reflecting collagen degradation (MMP degradation fragment of collagen III (C3M), MMP degradation fragment of collagen IV (C4M)) and representing collagen formation (N-terminal pro-collagen III peptide (PRO-C3), C-terminal pro-peptide of type V collagen (PRO-C5), and C-terminal pro-peptide of type VI collagen (PRO-C6) have previously been evaluated in preclinical models and humans [14, 17–19].

Several studies have shown that cART attenuates hepatic ECM remodelling in HIV and that levels of collagen neo-epitope formation fragments PRO-C3 and PRO-C4 become significantly decreased in cART-treated PWH [20]. Recently, we demonstrated that PRO-C3 reflects liver fibrosis and positively correlates with bilirubin, reduced platelet count and low albumin levels mirroring liver dysfunction in cART-treated PWH [21]. However, since PRO-C3 measurements do not differentiate between single stranded or cross-linked N-terminal pro-peptide strands, measuring cross-linked type III collagen pro-peptides (PC3X) could have additional diagnostic and prognostic potential for fibrogenesis in HIV disease. Recently, Jensen et al. showed that PC3X is associated with hepatocellular carcinoma (HCC) independent of AFP and provides diagnostic and prognostic value for HCC-patients [22]. PRO-C5 could be suitable for the non-invasive evaluation of portal hypertension in individuals with alcoholic cirrhosis [23].

PRO-C6, a fragment of Type VI collagen, the pro-peptide of which is also known as the hormone endotrophin, has been recently identified as biomarker of survival in individuals with liver cirrhosis and HCC [24]. The level of endotrophin assessed via PRO-C6 was able to separate healthy controls, noncirrhotic individuals and cirrhosis from HCC. Additionally PRO-C6 levels were higher in individuals with advanced fibrosis stage in non-alcoholic steatohepatitis (NASH) [25]. Endotrophin is an adipokine acting in adipose tissue. It mediates an elevation of pro-inflammatory cytokines and insulin resistance in many tissues [26].

So far, the significance of PC3X, PRO-C5 and PRO-C6 in regard to detection of fibrosis and steatosis in people with HIV has not been investigated yet. Therefore, the aim of this study was to analyse the ECM remodelling by assessing the levels of PC3X, PRO-C5, and PRO-C6 in serum samples from well-characterized PWH with respect to fibrosis and steatosis as measured by transient elastography using a Fibroscan with integrated controlled attenuation parameter (CAP).

## Methods

### Participants

We initiated a pilot study in 2015 and recruited 141 PWH at the outpatient department at the Bonn University Hospital. Some data from the PWH without HCV of this cohort has been previously published [18]. All PWH received cART following the regularly updated guidelines for antiretroviral treatment as recommended by the European AIDS Clinical Society (EACS); 9 individuals still had low-level detectable HIV viral load under treatment at the time of recruitment. 18 participants were anti-HCV positive (with chronic, ongoing HCV infection). Seven participants were anti-Hbc positive, of these, 4 had detectable Hbs-antigen.

Relevant alcohol consumption was excluded by questionnaire. An alcohol intake of above 30 g/week (for men) or 20 g/week (for women) was considered as relevant consumption and individuals with such were not included into the study. All participants underwent careful clinical examination, Fibroscan and CAP measurement. Furthermore, biochemical tests including blood count, alanine and aspartate aminotransferases, alkaline phosphatase, bilirubin, and y-glutamyltranspeptidase were analysed in fasting blood samples at the same day as clinical examination.

### Enzyme-linked immunoabsorbent assay

Markers of V and VI collagen formation, PRO-C5, and PRO-C6, and cross-linked type III collagen, PC3X, were analyzed in serum samples using specific enzyme-linked immunosorbent assays (ELISA). The ELISAs were performed by Nordic Bioscience A/S, Herlev, Denmark as described elsewhere [22, 23, 27].

### Diagnosis of HIV and viral hepatitis

HIV viral load was determined quantitatively with the Abbott RealTime m2000rt (Abbott Laboratories, Illinois, USA). This assay had a detection limit of 40 copies/mL. In addition, chronic viral hepatitis was examined via routine assays for hepatitis B surface antigen, HBV-DNA, HCV antibodies and HCV-RNA.

### Transient elastography and CAP measurement

We measured liver stiffness using a “Fibroscan 502 Touch” device equipped with an M probe (Echosens, Paris, France) and classified individuals as having no fibrosis when kPa values were below 7.1 and as clinical significant fibrosis when values were ≥ 7.1 [2]. In addition, hepatic steatosis was assessed via the controlled attenuation parameter (CAP) technology [28, 29]. This non-invasive technique measures the longitudinal attenuation of elastic waves in liver tissue at the centre frequency of the Fibroscan probe and yields results in dB/m that correlate to intrahepatic fat content [30]. We stratified PWH according to CAP values: no steatosis when CAP values were below 238 dB/m and presence of steatosis with values higher than 238 dB/m [29, 31]. Participants had the following grades of fibrosis: n = 116 had F1, n = 14 F2, n = 5 F3, n = 6 F4 and steatosis: 79 had no steatosis (S0), 22 had S1 steatosis, n = 19 had S2 and n = 21 had S3. Liver stiffness and CAP were obtained simultaneously in the same volume of liver parenchyma and represent the median of 10 measurements.

### Statistical methods

Differences in serum levels of PC3X, PRO-C5, and PRO-C6 between PWH-groups were analyzed using Pearson's goodness-of-fit chi^2^ test, paired Student t test and ANOVA as appropriate. Correlations between levels of PC3X, PRO-C5, and PRO-C6, liver stiffness and biochemical data were analyzed by using Spearman correlation coefficient. A Cox regression analysis was calculated to identify factors associated with fibrosis (liver stiffness ≥ 7.1 kPa) or steatosis (CAP ≥ 238 dB/m).

We constructed Receiver-operating characteristics to assess the accuracy of PC3X, PRO-C6 measurement and to determine the optimal cutoffs to differentiate between relevant or non-relevant fibrosis (cutoff 7.1 kPa). The optimal cutoff value was chosen at the point with the highest Youden’s index. The same calculation was performed for PRO-C5 but to determine the optimal cutoff to identify the presence of steatosis (cutoff 238 dB/m). Calculations and graphs were obtained with the SPSS statistics software (version 24) and GraphPad Prism 8.0 software package (GraphPad Prism, San Diego California, USA), respectively.

## Results

### Characteristics of study participants

**Fig. 1:**
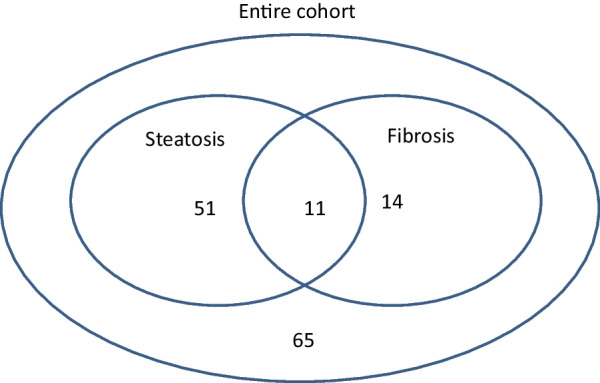
Study cohort. Venn-diagram illustrates the number of PWH (total = 114) with relevant steatosis (n = 51), fibrosis (n = 14) or both (n = 11). 65 participants had neither relevant steatosis nor fibrosis

We recruited 141 PWH whose median age was 47 years (range 24–72). All PWH received cART for at least one year at the date of enrolment and most participants (94%) had RNA levels below the detection limit. Only nine participants had a detectable low-level HIV viraemia, despite ongoing cART. Overall, our cohort consisted of 141 PWH of whom 51 (44.7%) had steatosis and 14 (12.3%) fibrosis and 11 (9.6%) both (Fig. 1).

### Levels of PC3X and PRO-C6 in serum of PWH reflect status of fibrosis

**Fig. 2:**
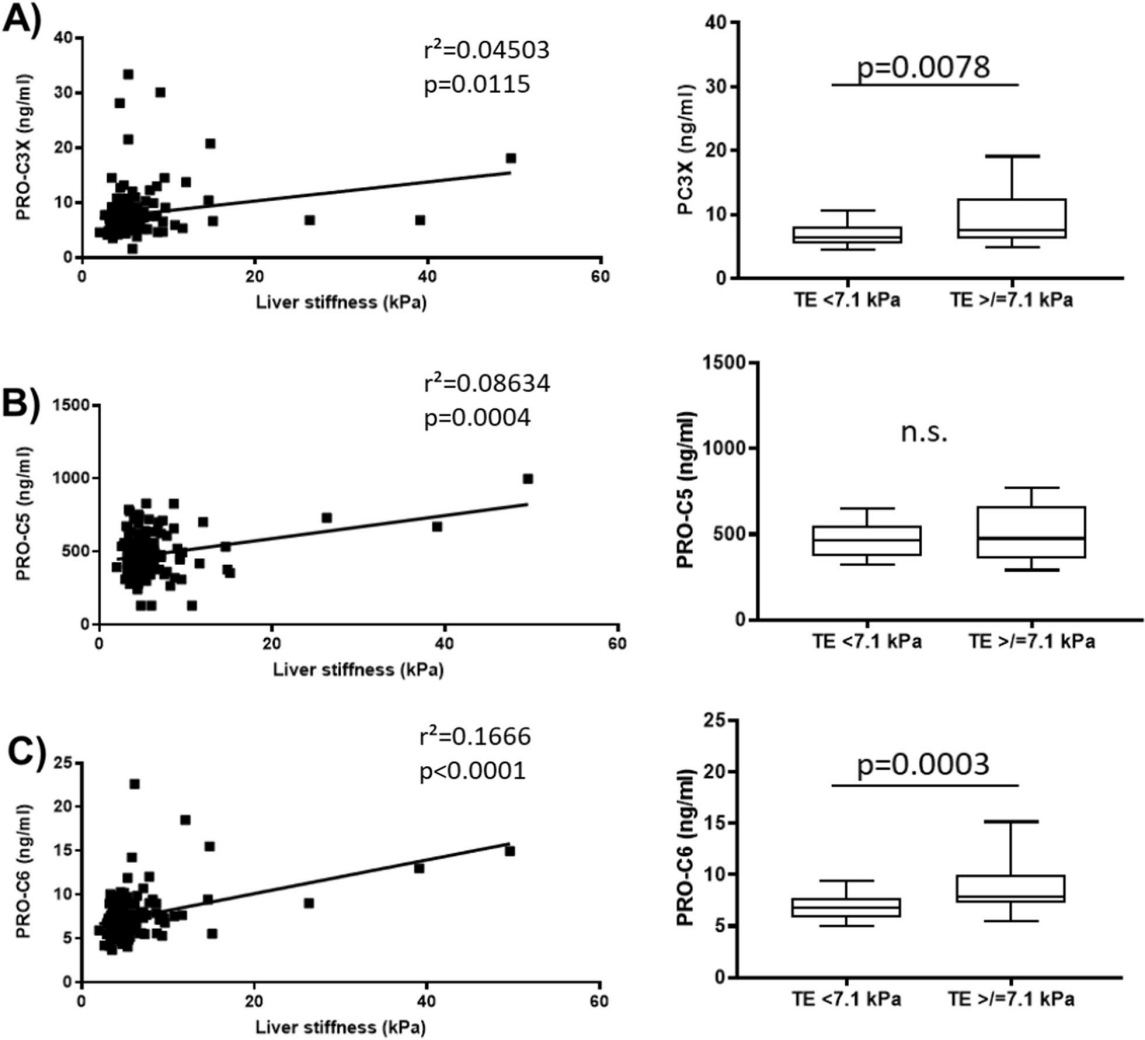
PC3X and PRO-C6 serum levels of PWH correlated with liver stiffness. **A** illustrates that serum levels of PC3X correlate with liver stiffness and are significantly higher in PWH with relevant fibrosis versus individuals without fibrosis. **B** demonstrates that serum levels of PRO-C5 correlate with liver stiffness, but levels are not different between PWH with relevant fibrosis versus individuals without fibrosis. **C** illustrates that serum levels of PRO-C6 correlate with liver stiffness and are significantly higher in PWH with relevant fibrosis versus individuals without fibrosis

Study participants were divided into two groups based on liver stiffness (Table 1): first group consisted of 116 PWH (82.3%) who had no clinical significant fibrosis (stiffness < 7.1 kPa) and second group of 25 individuals (17.7%) with significant hepatic fibrosis (stiffness ≥ 7.1 kPa). Comparing both groups, PWH with significant fibrosis were older (p = 0.004) and had markedly higher serum levels of AST (p = 0.037) than PWH without fibrosis. In addition, number of thrombocytes were significantly lower in PWH with fibrosis compared to participants without fibrosis (p = 0.0001). Levels of PC3X and PRO-C6 were significantly (p < 0.01) higher in individuals with fibrosis > 7.1 kPa (Table 1, Fig. 2A, C).

**Table 1:** Clinical and demographical characteristics of PWH according to liver stiffness

	PWH (all)	PWH without clinical significant fibrosis (Stiffness < 7.1 kPa)	PWH with clinical significant fibrosis (Stiffness ≥ 7.1)	P-value
Total number of participants n (%)	141 (100)	116 (82.3)	25 (17.7)	N.a
Age (years)	47 (24–72)	46 (24–72)	52 (36–67)	**0.004**
Sex (male/female)	120/21	100/20	20/1	N.s
BMI (kg/cm2)	24 (19–36)	24 (20–36)	24 (19–32)	N.s
AST (U/L)	23 (9–93)	22 (9–65)	27 (11–93)	**0.037**
ALT (U/L)	29 (10–110)	29 (10–103)	33 (10–110)	N.s
GGT (U/L)	46 (16–302)	45 (16–302)	60 (26–248)	N.s
Thrombocytes	206 (46–400)	213 (123–400)	167 (46–259)	**0.0001**
Transient elastography (kPa)	4.9 (2.0–49.6)	4.6 (2.0–6.9)	9.3 (7.1–49.6)	N.a
Steatosis CAP (dB/m)	232 (100–378)	231 (100–378)	236 (101–364)	N.s
Cholinesterase	13,264 (7168–24,175)	13,797 (7168–22,233)	11,028 (7567–24,175)	N.s
PC3X (ng/ml)	6.7 (1.5–33.3)	6.5 (1.5–33.3)	7.5 (4.6–30.1)	**0.0078**
PRO-C5 (ng/ml)	465.0 (130.0–998.4)	463 (130.0–828.5)	476.6 (130.0–998.4)	N.s
PRO-C6 (ng/ml)	6.9 (3.7–22.6)	6.8 (3.7–22.6)	7.9 (5.3–18.5)	**0.0003**
Anti-HCV-positive (%)	18 (12.8)	11 (9.5)	7 (28.0)	N.s
Hbs-Ag positive (%)	4 (2.8)	3 (2.6)	1 (4.0)	N.s

All values are given as median and range. *ALT* alanine aminotransferase, *AST* aspartate aminotransferase, *BMI* Body-mass index, *CAP* Controlled attenuation parameter, *GGT* γ–glutamyl transferase, *n.a.* not applicable, *n.s.* not significant

Significant p-values are shown in bold

Since we aimed to study whether PC3X and PRO-C6 can be used to distinguish if clinical significant fibrosis (> 7.1 kPa) is present or not, we looked at two groups (with and without fibrosis). However, both markers showed an increase with advanced fibrosis. For PC3X: F1 median 6.5 (range 1.5–33.3), F2 median 7,503 (range 4.6–30.1), F3 median 9.1 (range 5.3–14.5), F4 median 8.6 (range 6.6–20.7). For PRO-C6: F1 median 6,8 (range 3.7–22.6), F2 median 7,8 (5.3–12.0), F3 median 7,5 (6.8–18.5), F4 median 11,21 (5.53–15.48). In contrast, PRO-C5 did not differ between individuals with clinical significant fibrosis and non-fibrosis (Fig. 2B right).

To determine factors which predict the presence of fibrosis (Fibroscan value ≥ 7.1 kPa) in PWH we calculated a cox logistic regression. Univariate regression analysis identified PC3X and PRO-C6 in addition to ALT, AST, and platelets as potential factors for relevant fibrosis. PRO-C5 was not statistically significant (Table 2). When we calculated a multivariate analysis comprising all parameters significant in univariate analysis, only PC3X, AST, and platelets remained statistically significant (Table 2). We further determined cut-off values, sensitivity, and specificity of PC3X and PRO-C6. By calculating ROC curves, we determined a value of 7.13 ng/mL of PRO-C6 to identify a clinical significant fibrosis (≥ 7.1 kPa), and for PC3X a value of 6.6 ng/mL as cut-off.

**Table 2:** Regression analysis to identify factors to predict the presence of fibrosis (≥ 7.1 kPa)

Parameter	OR	95% CI	p-value
Univariate analysis			
PCX3	1.116	1.025–1.215	**0.012**
PRO-C5	–	–	0.274
PRO-C6	1.272	1.076–1.502	**0.005**
ALT	1.027	1.005–1.048	**0.015**
AST	1.051	1.018–1.086	**0.003**
yGT	1.007	1.000–1.014	**0.059**
Platelets	0.976	0.965–0.988	**0.001**
Age	1.050	1.000–1.103	**0.051**
BMI	–	–	0.820
Multivariate analysis			
PC3X	1.127	1.003–1.265	0.04
AST	1.051	1.002–1.102	0.028
Platelets	0.970	0.954–0.987	0.001

Significant p-values are shown in bold

PRO-C6 had a high sensitivity of 80% and specificity of 78%, while sensitivity of PC3X was 72% with a specificity of 72%.

In order to identify possible confounding factors, we calculated if duration of cART had an influence on ECM biomarker levels. Duration of cART did not correlate to levels of PC3X (p = 0.886), PRO-C5 (p = 0.339) or PRO-C6 (p = 0.098). Also, detectable HIV viral load did not influence ECM biomarker levels. Additionally, immunological parameters such as CD4, CD8 or CD4/CD8 ratios had no impact on fibrosis or the levels of ECM markers. We further investigated the role of confection with viral hepatitis. Unlike HCV, a history of HBV infection (positive anti-Hbc or positive HbsAg) did not influence levels of PC3X, PRO-C5 and PRO-C6. However, a history of, or ongoing HCV replication (positive anti-HCV), showed a weak correlation to levels of PRO-C6 (p = 0.45). We therefore re-calculated sensitivity and specificity of PRO-C6 for all PWH without HCV resulting in a PRO-C6 value of 7.2 ng/ml to identify a clinical significant fibrosis (≥ 7.1 kPa) with a sensitivity of 76.9% and specificity of 65.0%.

Overall, PRO-C6 had a slightly higher sensitivity (80%) and specificity (78%) in the whole cohort including co-infected individuals but was comparably sufficient in PWH without HCV (as shown above).

### Relationship between PC3X, PRO-C5, PRO-C6 values and steatosis

When we stratified PWH according to their CAP values into two additional groups (Table 3): In 79 individuals (56.0%) no hepatic steatosis was detected (CAP < 238 dB/m; group 1), whereas 62 participants (44.0%) were allocated to have probable fatty liver disease (CAP ≥ 238 dB/m; group 2). Overall, PWH with steatosis had higher BMI than individuals without steatosis (p = 0.010) and tended to exhibit higher levels of liver enzymes (AST, ALT, GGT) than PWH without steatosis (Table 3). We did not find significant differences regarding age and sex. Here, no significant differences were detected for PC3X and PRO-C6, however individuals with steatosis had significantly higher serum levels of PRO-C5.

**Table 3:** Clinical and demographical characteristics of PWH stratified by liver steatosis

	PWH (all)	PWH without presence of steatosis (CAP < 238 dB/m)	PWH with steatosis (CAP ≥ 238 dB/m)	P-value
Total number of participants n (%)	141 (100)	79 (56.0)	62 (44.0)	N.a
Age (years)	47 (24–72)	47 (24–72)	47 (30–69)	N.s
Sex (male/female)	120/21	65/14	55/7	N.s
BMI (kg/cm2)	24 (19–36)	23 (19–33)	25 (20–36)	**0.010**
AST (U/L)	23 (9–93)	23 (9–65)	24 (13–93)	N.s
ALT (U/L)	29 (10–110)	29 (10–99)	31 (12–110)	**0.015**
GGT (U/L)	46 (16–302)	45 (17–301)	51 (16–302)	N.s
Thrombocytes	206 (46–400)	203 (46–400)	212 (78–342)	N.s
Transient elastography (kPa)	4.9 (2.0–49.6)	4.7 (22.0–49.0)	5.3 (3.1–26.3)	N.s
Steatosis CAP dB/m)	232 (100–378)	210 (100–237)	272 (238–378)	N.a
Cholinesterase	13,264 (7168–24,175)	12,501 (7168–22,233)	14,824 (7265–24,175)	N.s
PC3X (ng/ml)	6.7 (1.5–33.3)	6.5 (3.4–33.3)	6.8 (1.5–21.5)	N.s
PRO-C5 (ng/ml)	465 (130.0–998.4)	443.9 (130.0–998.4)	503.0 (130.0–825.4)	**0.014**
PRO-C6 (ng/ml)	6.9 (3.7–22.6)	6.9 (3.7–22.6)	7.0 (4.6–18.5)	N.s
Anti-HCV-positive (%)	18 (12.8)	11 (13.9)	7 (11.3)	N.s
Hbs-Ag positive (%)	4 (2.8)	1 (1.3)	3 (4.8)	N.s

All values are given as median and range

*ALT* alanine aminotransferase, *AST* aspartate aminotransferase, *BMI* Body-mass index, *CAP* Controlled attenuation parameter, *GGT* γ–glutamyl transferase, *n.a.* not applicable, *n.s.* not significant

Significant p-values are shown in bold

Serum levels of PRO-C5 were correlated to steatosis as determined by CAP measurement (Additional file 1: Fig. S1A-C left). In contrast, PC3X and PRO-C6 did not differ between individuals with and without relevant steatosis (Additional file 1: Fig. S1A and C).

For PRO-C5 we calculated a value of 6.5 ng/ml as cut-off to identify relevant steatosis (≥ 238 dB/m). PRO-C5 had a high sensitivity of 100% but low specificity of 51%.

Next, we calculated a Cox logistic regression model to identify prognostic factors for relevant steatosis (CAP ≥ 238 dB/m). However, only univariate regression confirmed PRO-C5 in addition to ALT and BMI as potentially prognostic factors for steatosis, while PC3X and PRO-C6 as well as all other factors (AST, yGT, platelets, age) were not statistically significant (Additional file 1: Table S1). Moreover, none of the parameters remained statistically significant in multivariate analysis.

Finally, neither PC3X, PRO-C5 nor PRO-C6 correlate with parameters of liver inflammation and parameters of liver function, such as albumin, ALT, AST, cholinesterase, and platelet count when calculated in a Spearmans correlation analysis. We did not have any longitudinal data available to determine the role of PC3X, PRO-C5 or PRO-C6 as prognostic markers for disease progression.

## Discussion

This study is the first to analyse the association between collagen remodelling of type III, V and VI collagen assessed by PC3X, PRO-C5, PRO-C6 and hepatic fibrosis and steatosis in PWH treated with cART. PWH are at a high risk for liver related complications and elevated liver enzymes [1, 32, 33]. Thus, liver disease has emerged as a severe challenge and contributes to 7–14% of acquired immune deficiency syndrome (AIDS)‐related deaths [34]. It was shown that hepatic steatosis is highly prevalent among PWH receiving cART [3]. Furthermore, there is an important need to identify PWH who have a spontaneous fibrosis regression. Therefore, non-invasive screening methods could help to early identify changes in liver stiffness or liver fat content in PWH, since liver biopsy can be hampered by sampling error and severe complications [35] and transient elastography is not available in every health care centre.

In our study we demonstrated that in PWH formation of cross-linked type III collagen (PC3X) and endotrophin (PRO-C6) correlate with grade of fibrosis, but not with steatosis. In addition to ALT levels and platelet count, PC3X was confirmed as possible prognostic factor to identify clinically significant fibrosis via cox regression analysis. Jensen et al. described that PC3X was associated with HCC and thus provides diagnostic and prognostic value for HCC diseased individuals [22]. Since HCC most likely occurs in cirrhotic liver parenchyma, it is possible that PC3X has a strong diagnostic value in individuals with advanced/progressive liver fibrosis; however, a value of 6.6 ng/ml enabled to identify fibrosis with only a sensitivity of 72%. Therefore, it seems a useful parameter for the detection of fibrosis in PWH. PRO-C6 may have prognostic potential to predict survival in HCC. PRO-C6 was described to be significantly higher in individuals with advanced fibrosis stage 3–4 than those with fibrosis stage 0–2 in a cohort of individuals with non-alcoholic liver disease (NAFLD) [25]. Although, our cohort has a low prevalence of advanced fibrosis, PRO-C6 had a high sensitivity of 80% and specificity of 78% to detect fibrosis in PWH and consequently may be superior to PC3X for this purpose.

To our knowledge, we are the first to investigate the role of PC3X and PRO-C6 in PWH. Since PC3X is a more late-stage fibrosis marker, due to assessing type III collagen cross-linking and hence tissue stiffness, this may explain, why PC3X correlates to liver fibrosis only but not to parameters of liver function in the present PWH-cohort, that contains only few participants with clinical significant fibrosis or elevated liver enzymes.

Interestingly, we found a correlation of PRO-C5 with hepatic steatosis. This was a novel finding, as a role of PRO-C5 in hepatic steatosis has not been described yet. PRO-C5 has only been described in the context of advanced fibrosis so far and is a marker responsible for type I and III collagen fibrillation. For example, Leeming et al. report that plasma PRO-C5 levels highly correlate to portal hypertension in individuals with alcoholic cirrhosis, and PRO-C5 may be suitable for the non-invasive evaluation of portal hypertension in individuals with cirrhosis [23]. Furthermore, PRO-C5 was elevated and prognostic for transplant-free survival in people with primary sclerosing cholangitis as compared to ulcerative colitis [36]. None of these studies describes a role of PRO-C5 in hepatic steatosis. However, our cohort contains only very few individuals with advanced steatosis, therefore, it would be interesting to validate its role in a cohort of individuals with higher grades of steatosis (> S3). Of note, PRO-C5 had a high sensitivity of 100% to identify hepatic steatosis and it might serve as a non-invasive marker to detect early stages of hepatic steatosis. Due to its low specificity, it could be used as a first screening test in centres where CAP measurement is not available. When PRO-C5 measurements suggest possible hepatic steatosis, further tests such as CAP and ultrasound should be used to clarify the diagnosis. Regarding fibrosis, we found that PRO-C5 does not significantly correlate with fibrosis, therefore we think that PRO-C5 might only be suitable to differentiate between F4 fibrosis and low fibrosis and is less applicable in early stages as seen in our cohort.

Since transient elastography analysis is a well-established technique for the non-invasive detection of fibrosis and steatosis [2, 37–39], we conclude that the correlation to PC3X and PRO-C6 levels may allow to draw a conclusion about the presence of fibrosis in PWH. Since all the collagens analysed in this study are interstitial matrix markers, we suggest them to be particularly relevant in late-stage fibrosis whereas the type IV collagen markers and other markers of basement membrane turnover such as PRO-C4 may be more suited for individuals with early-stage liver fibrosis. Since PC3X and PRO-C6 seems to have a prognostic role in HCC, it would be interesting to evaluate this marker in longitudinal data of PWH to see how levels of PC3X correlate to HCC development in these individuals.

The main limitation of this study is its low number of participants and the retrospective nature of the study. Since all treated PWH have liver enzyme levels similar to healthy participants, this study allows only limited conclusion about the usefulness of these markers in treated PWH. In addition, its cross-sectional design does not allow evaluating the dynamics of risk factors of severe hepatic fibrosis or steatosis over a time. The study represents a real-life study cohort. Additional supporting analysis, such as magnetic resonance tomography imaging to quantify fibrosis and steatosis were not available.

In conclusion, this study demonstrates that liver fibrosis is reflected by PC3X and with a lesser extent by PRO-C6 levels in PWH. These markers may be used to monitor people at risk of fibrosis and to non-invasively detect higher grades of fibrosis. PRO-C5 could be a parameter for the early detection of hepatic steatosis in PWH.

## Supplementary Information

**Additional file 1: Figure S1.** PRO-C5 serum levels of PWH correlated with steatosis. **Table S1.** Regression analysis for independent factors for the presence of steatosis.

## Data Availability

All data generated or analysed during this study are included in this published article and its Additional files.
