# Supplementary material for: Circulating levels of endotrophin and cross-linked type III collagen reflect liver fibrosis in people with HIV
Source: BMC Infect Dis. 2023 Jan 24;23:52. doi: 10.1186/s12879-023-08000-w (PMC9872428; doi:10.1186/s12879-023-08000-w)
Supplement: Supplementary file 1 — Additional file 1: Figure S1. PRO-C5 serum levels of PWH correlated with steatosis. Table S1. Regression analysis for independent factors for the presence of steatosis. [file 12879_2023_8000_MOESM1_ESM.docx]

**Suppl. Figure 1**

**A)**

**B)**

**C)**

n.s.

r²=0.03544

p=0.0254

n.s.

p=0.0134

n.s.

n.s.

**Additional file 1:** **Figure S1. PRO-C5 serum levels of PWH correlated with steatosis.**

Figures **A and C** illustrate that serum levels of PC3X and PRO-C6 do not correlate with steatosis in PWH.

Figure **B** demonstrates that serum levels of PRO-C5 correlate with steatosis and are significantly higher in HIV-patients with relevant steatosis versus patients without steatosis.

**Additional file 1: Table S1**

| Regression analysis: to predict the presence of steatosis (≥238 dB/m) | | | |
| --- | --- | --- | --- |
| Univariate analysis | | | |
| **Parameter** | **OR** | **95%-CI** | **p-value** |
| PCX3 | -------- | ------------------------------ | 0.340 |
| PRO-C5 | 1.003 | 1.001-1.005 | **0.016** |
| PRO-C6 | -------- | ------------------------------ | 0.758 |
| ALT | 1.025 | 1.005-1.045 | **0.015** |
| AST | -------- | ------------------------------ | 0.060 |
| yGT | -------- | ------------------------------ | 0.088 |
| Platelets | -------- | ------------------------------ | 0.408 |
| Age | -------- | ------------------------------ | 0.182 |
| BMI | 1.200 | 1.041-1.384 | **0.012** |

**Additional file 1: Table S1: Regression analysis for independent factors for the presence of steatosis.**
